# Endonuclease G takes part in AIF-mediated caspase-independent apoptosis in *Mycobacterium bovis*-infected bovine macrophages

**DOI:** 10.1186/s13567-018-0567-1

**Published:** 2018-07-18

**Authors:** Alejandro Benítez-Guzmán, Lourdes Arriaga-Pizano, Julio Morán, José A. Gutiérrez-Pabello

**Affiliations:** 1 Laboratorio de Investigación en Tuberculosis Bovina, Departamento de Microbiología e Inmunología, Facultad de Medicina Veterinaria y Zootecnia, Universidad Nacional Autónoma de México, Mexico City, Mexico; 2 Unidad Médica de Investigación en Inmunoquímica, Hospital Siglo XXI, IMSS, Mexico City, Mexico; 3 División de Neurociencias, Instituto de Fisiología Celular, Universidad Nacional Autónoma de México, México City, Mexico

## Abstract

*Mycobacterium bovis*, the causative agent of bovine tuberculosis encodes different virulence mechanisms to survive inside of host cells. One of the possible outcomes in this host–pathogen interaction is cell death. Previous results from our group showed that *M. bovis* induces a caspase-independent apoptosis in bovine macrophages with the possible participation of apoptosis inducing factor mitochondria associated 1 (AIFM1/AIF), a flavoprotein that functions as a cell-death regulator. However, contribution of other caspase-independent cell death mediators in *M. bovis*-infected macrophages is not known. In this study, we aimed to further characterize *M. bovis*-induced apoptosis, addressing Endonuclease G (Endo G) and Poly (ADP-ribose) polymerase 1 (PARP-1). In order to accomplish our objective, we infected bovine macrophages with *M. bovis* AN5 (MOI 10:1). Analysis of *M. bovis*-infected nuclear protein extracts by immunoblot, identified a 15- and 43-fold increase in concentration of mitochondrial proteins AIF and Endo G respectively. Interestingly, pretreatment of *M. bovis*-infected macrophages with cyclosporine A, a mitochondrial permeability transition pore inhibitor, abolished AIF and Endo G nuclear translocation. In addition, it also decreased macrophage DNA fragmentation to baseline and caused a 26.2% increase in bacterial viability. We also demonstrated that PARP-1 protein expression in macrophages did not change during *M. bovis* infection. Furthermore, pretreatment of *M. bovis*-infected bovine macrophages with 3-aminobenzamide, a PARP-1 inhibitor, did not change the proportion of macrophage DNA fragmentation. Our results suggest participation of Endo G, but not PARP-1, in *M. bovis*-induced macrophage apoptosis. To the best of our knowledge this is the first report associating Endo G with caspase-independent apoptosis induced by a member of the *Mycobacterium tuberculosis* complex.

**Electronic supplementary material:**

The online version of this article (10.1186/s13567-018-0567-1) contains supplementary material, which is available to authorized users.

## Introduction

Apoptosis is a cell death mechanism involved in the regulation of physiological processes to maintain homeostasis in mammals. However, it is also implicated in the pathogenesis of different disorders like cancer, as well as, bacterial, viral and parasitic infections [1–3]. Apoptosis takes place through two general pathways: one that is associated with cell receptors (extrinsic) and a second one that includes cytoplasmic organelles (intrinsic) [4].

In mycobacterial infections, apoptosis is one of the possible outcomes of the host–pathogen interaction [5]. Results from different research groups have demonstrated that apoptosis induction by mycobacterial species use both aforementioned pathways, although in most cases it is a caspase-dependent process [6, 7]. Components of the bacterial cell wall and secretion proteins, as well as host cell metabolites, such as nitric oxide (NO), have been identified as inducers of apoptosis [8]. Different studies have confirmed the association between reduced bacterial viability and apoptosis, therefore it is considered a host-protective response [9].

Mitochondria drive apoptosis through the intrinsic pathway [10]. BH1–BH3 domain-containing Bcl-2 family proteins; Bax and Bak, are proteolipid pore forming proteins, responsible for mitochondrial outer membrane permeabilization (MOMP) [11]. MOMP leads to the release of apoptosis inducing proteins, such as cytochrome C (caspase-dependent) and apoptosis inducing factor mitochondria associated 1 (AIFM1/AIF) or Endonuclease G (Endo G) (caspase-independent) [11]. Endo G is a mitochondrial nuclease that under regular conditions plays a role in mitochondrial DNA replication, however its nuclease activity has also been identified in cells undergoing an apoptotic process [12, 13]. Upon different stimuli, Endo G is released from mitochondria and translocated to the nucleus where it cleaves chromatin DNA into nucleosomal fragments independently of caspases [14]. Parthanatos is a caspase-independent cell death pathway that encompasses activation of the DNA repair protein Poly (ADP-ribose) polymerase-1 (PARP-1), accumulation of PAR polymers in the cytoplasm, as well as AIF release from mitochondria and translocation to the nucleus [15, 16]. Previous results from our group showed that *Mycobacterium bovis* induces a caspase-independent apoptosis in bovine macrophages with a possible participation of AIF and independent of NO production [17, 18]. In addition, we observed mitochondrial depolarization in macrophages treated with a *M. bovis* protein extract [18]. However, contribution of other caspase-independent cell death mediators in *M. bovis*-infected macrophages is not known. In this study, we aimed to further characterize *M. bovis*-induced apoptosis, addressing the involvement of Endo G and PARP-1 presence in the nuclei of infected macrophages.

Here we report, for the first time, that Endo G nuclear translocation occurs during caspase-independent apoptosis induced by *M. bovis*. We also demonstrate that PARP-1 protein expression in macrophages did not change during *M. bovis* infection and that PARP-1 inhibition did not change the proportion of macrophage DNA fragmentation. Our results suggest participation of AIF and Endo G, but not PARP-1, in *M. bovis* induced apoptosis.

## Materials and methods

### Macrophage culture

Venous peripheral blood was obtained from healthy adult cattle, from a tuberculosis-free herd, housed in the facilities of the Research and Teaching Center (CEPIPSA) of the Universidad Nacional Autónoma de México (UNAM). Macrophages were obtained from peripheral blood mononuclear cells (PBMC) by the method of Stich et al. [19] with slight modifications. Blood was collected from the jugular vein into 60-mL syringes containing acid-citrate-dextrose solution and was centrifuged at 1000 × *g* for 30 min. Buffy coats were diluted in 30 mL citrated PBS, then layered onto 15 mL Percoll (Pharmacia, Uppsala, Sweden) at a specific density of 1.077, and centrifuged at 1200 × *g* for 25 min. PBMC were then removed from the interface between the plasma and Percoll solution, pooled, diluted in 50 mL of citrated PBS, and centrifuged at 500 × *g* for 15 min. The cell pellets were then washed three times with citrated PBS at 500 × *g* for 10 min, suspended in RPMI (Gibco, New York, USA) supplemented with 5 mM l-glutamine (Gibco), 5 mM non-essential aminoacids and 5 mM sodium pyruvate (Gibco) containing 4% autologous serum (CRPMI) to facilitate adherence and cultured overnight at 37 °C and 5% CO_2_. Non-adherent cells were then removed by three washes with prewarmed PBS, and adherent monocytes were cultured just as described previously in CRPMI plus 12% autologous serum for 12 days until they differentiated to macrophages. Purity of macrophages was ≥ 88% as determined by FACS analysis (anti CD14 mAb, FITC conjugated; Miltenyi). Flasks were chilled on ice for 40 min and macrophages were harvested by repeatedly pipetting gently.

### *Mycobacterium bovis* infections

*Mycobacterium bovis* AN5 strain was grown at 37 °C under shaking conditions in Middlebrook 7H9 broth with 0.05% Tween 80 and supplemented with 10% of OADC enrichment (Becton–Dickinson, Cockeysville, MD, USA) for 19 days to reach the mid-log phase of growth. Bacteria were suspended in CRPMI, passed twice through a 27-gauge needle and stored at −80 °C in 1 mL aliquots until further use. Inoculums were titrated by plating CFU serial dilutions on Middlebrook 7H11 medium (Difco Laboratories, Detroit, MI, USA) plus 10% of OADC. An aliquot of 1.2 × 10^6^ macrophages were infected in cell culture flasks of 25 cm^2^ with *M. bovis* at a multiplicity of infection (MOI) of 10:1 at 37 °C for 4, 8 and 16 h. For some experiments, monolayers were pre-incubated with 1 µM Cyclosporine A (Sigma, Mo, USA) or 50 µM 3-aminobenzamide (3-ABA) (Merck, Boston, USA) 2 h before infection, cultures were maintained in a humidified atmosphere with 5% CO_2_.

### Caspase 3 activity assay

Fluorogenic caspase 3 substrate Ac-YVAD-AMC (Peptide Institute Inc., Osaka, Japan) was used to identify caspase activation. An aliquot of 1 × 10^6^ macrophages were harvested in a reaction buffer containing 100 mM HEPES, 10% (w/v) sucrose, 0.1% (w/v) 3-[(3-cholamidopropyl)-dimethylammonio]-1-propanesulfonate (CHAPS), 10 mM DTT, protease inhibitors (Biovision, California, USA) and triton 1%. After harvesting, caspase activity was detected by a fluorogenic assay as described by Thornberry [20]. A total of 200 µg of cell homogenates were placed in 96 dark ELISA plates with 25 µM of fluorogenic substrate. Emission fluorescence in the 380/430–460 nm range was determined for 20 min using the Synergy HT Bio Tek microplate reader. Caspase activity was expressed as, fluorescence produced per minute during 15 min per 200 µg of protein and presented as mean ± standard deviation from two independent experiments. Differences among positive control and cellular lysates from *M. bovis*-infected macrophages at different time points were tested for statistical significance by one-way ANOVA. All tests were performed using SPSS18 software for windows. A *p* value equal or less than 0.05 was considered to be statistically significant.

### Nuclear isolation

Nuclei from 1.2 × 10^6^ infected macrophages were resuspended in sucrose buffer with NP40 [0.32 M sucrose, 3 mM CaCl_2_, 0.1 mM EDTA, 10 mM Tris–HCl, 1 mM DTT and proteases inhibitors (Biovision, CA, USA)] and centrifuged 500 × *g* for 5 min. Supernatant was collected and 0.22 V/V of cold cytoplasmic extraction buffer (0.15 M HEPES pH7, 0.7 M KCl, .015 M MgCl_2_) was added. Preparations were centrifuged at 12 000 × *g* for 15 min and supernatants were collected and stored at −80 °C, meanwhile pellets were resuspended in sucrose buffer without NP40 and quantified for use in Western blot (WB).

### Antibodies and Western blot

In each experiment, a total of 20 μg of nuclear proteins from infected macrophages were separated by SDS_PAGE at 10% and transferred to PVDF membranes. AIF and Endo G were detected by means of primary polyclonal antibodies, anti-AIF and anti-Endo G (Cell signaling, New England, USA), and secondary antibodies anti-rabbit/HRP (Cell signaling). The control load was monitored with an anti-Lamin A/C (Cell signaling). PARP-1 was detected from macrophage whole lysate (1.2 × 10^6^ cells) with Laemmli buffer, and proteins were separated by SDS-PAGE 8% and transferred to PVDF membranes, detection was accomplished by means of primary polyclonal antibodies anti-PARP-1 (Cell signaling) and anti-rabbit/HRP as a secondary antibody. The control load was monitored with an anti-Tubulin antibody (Cell signaling). All the blots were incubated overnight with primary antibodies diluted 1:1000, after three washes, secondary antibodies diluted 1:10 000 were incubated for 1 h and ECL (Femto west supersignal Pierce, Rockford, USA) development was accomplished with CL-Xposure film (Thermo scientific, Mo, USA) exposure. Densitometry analysis was performed using Image studio software of LICOR. The results were obtained by comparing the ratio, protein of interest/control load, and were expressed as a fold increase in relation to the negative control. The results are presented as mean ± standard deviation from three independent experiments. Differences among negative control and nuclear extracts of *M. bovis*-infected macrophages at different times were tested for statistical significance by one-way ANOVA. All tests were performed using SPSS18 software for windows. A *p* value equal or less than 0.05 was considered to be statistically significant.

### DNA fragmentation

Macrophages were fixed with paraformaldehyde 1% and stained using the ApoBrdU-TUNEL assay kit (Invitrogen, OR, USA) according to the manufacturer’s protocols. Data was acquired using a FACS Aria flow cytometer (Becton and Dickinson Bioscience, San Jose, Cal, USA) and analyzed with Infinicyt software V 1.5 (Cytognos, Salamanca, Spain). Determination of fold increase was based on median fluorescence intensity (MFI) values for BrdU-FITC fluorescence in relation to the negative control. The results are presented as mean ± standard deviation from three independent experiments. Differences among negative control and *M. bovis*-infected macrophages at different conditions were tested for statistical significance by one-way ANOVA followed by Tukey’s or Dunnett’s multiple comparison tests. All tests were performed using SPSS18 software for windows. A *p* value equal or less than 0.05 was considered to be statistically significant.

### Bactericidal assay

The bactericidal assay was performed as described by Qureshi et al. [21], with some modifications. Macrophage monolayers in tissue culture Terasaki HLA plates (Nunc, Roskilde, Denmark) were infected with *M. bovis* at a multiplicity of infection (MOI) of 10:1, centrifuged at 200 × *g* for 10 min and incubated at 37 °C in a humidified atmosphere with 5% CO_2_ for 4 h. After waiting the allotted time for phagocytosis (4 h), the cells were washed 5 times with CRPMI and 12% of autologous serum to remove the extracellular bacteria, and incubated again at 37 °C. This was considered the starting time to evaluate bacterial intracellular replication. The cells were harvested just after removal of extracellular bacteria and 24 h later for analysis. Bacterial phagocytosis was calculated by plating serial dilutions of the live intracellular bacteria released from the macrophages after treatment with 0.5% Tween 20. The total number of colony forming units was divided by the total number of macrophages to obtain an average bacterial concentration per macrophage. Bacterial growth was calculated as the ratio of the total number of intracellular bacteria at the end of the assay in relation to the total number of bacteria at the start of the assay expressed as percent. The results are the average of three independent experiments. Differences among treatments were tested for statistical significance by one-way ANOVA. All tests were performed using SPSS18 software for windows. A *p* value equal or less than 0.05 was considered to be statistically significant.

## Results

### *Mycobacterium bovis* AN5 induces DNA fragmentation with AIF translocation to macrophage nuclei in a caspase-independent fashion

**Figure 1:**
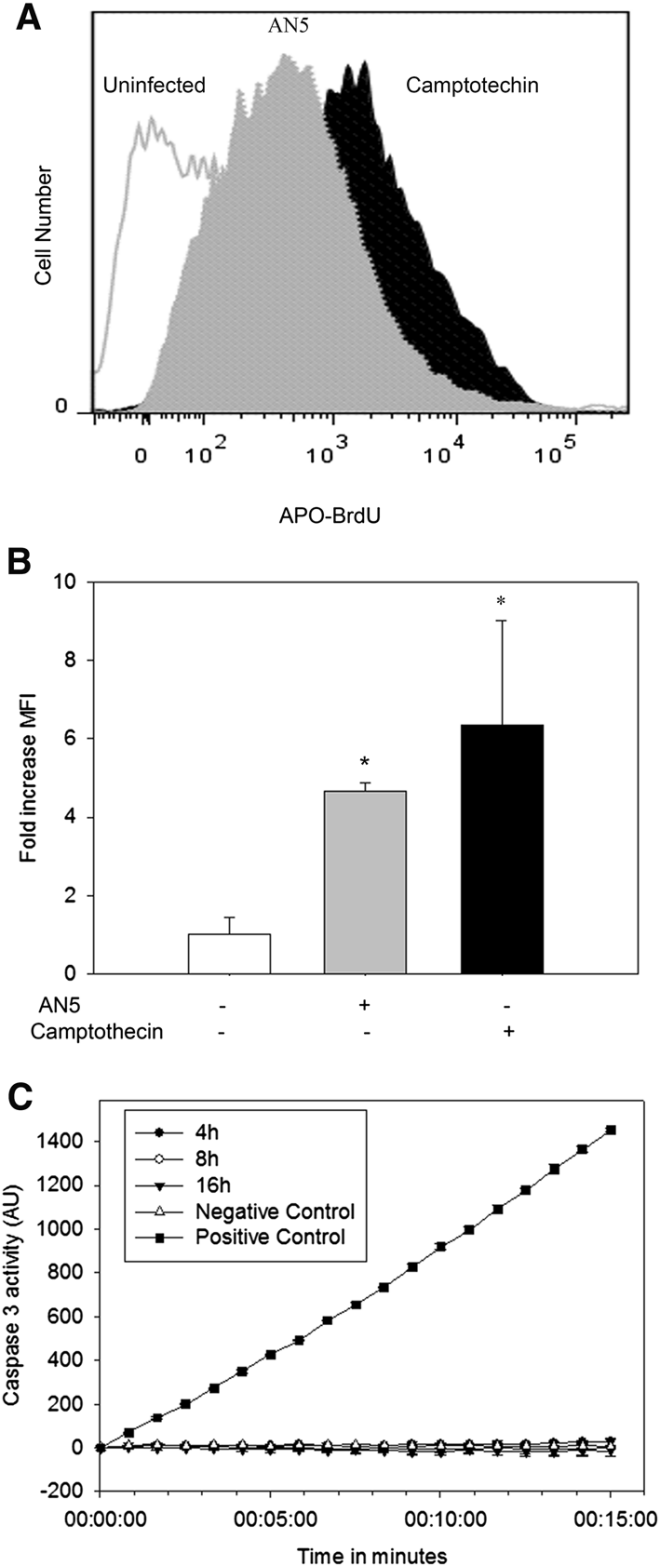
***Mycobacterium bovis*** **AN5 strain induces caspase-3-independent DNA fragmentation in bovine macrophages.** Bovine macrophages (1.2 × 10^6^) were either infected (MOI 10:1) with *M. bovis* AN5 strain or camptothecin-treated (25 µg/mL) during 16 h. **A** After infection, DNA fragmentation was analyzed by TUNEL (BrdU-FITC), the experiment is a representative FACS plot of TUNEL result. The results are expressed as total cell counts versus FITC signal in log_10_ scale. **B** Quantification of DNA fragmentation in A, bars represent the mean fluorescence intensity (MFI) fold change of three independent experiments of *M. bovis*-infected and camptothecin-treated macrophages with respect to uninfected cells. One-way ANOVA showed significant variation of MFI in infected versus uninfected cells (*P* < 0.05). **C** Caspase activity was evaluated by a fluorogenic technique using a caspase three specific substrate (Ac-YVAD-AMC). Macrophages were incubated under each of the following conditions: camptothecin (25 µg/mL) (positive control), CRPMI medium (negative control), *M. bovis* (MOI, 10:1) 4, 8, and 16 h. The results are expressed as fluorescence emitted per minute during 15 min per 200 µg of protein and are mean ± standard deviation from two independent experiments, each with three internal replicas. There was a statistical significance difference (*p* ≤ 0.05) as early as 3 min after the substrate addition. Statistical difference persisted across the experiment.

This is the first report demonstrating that the *M. bovis* reference strain AN5 is competent to induce apoptosis. Bovine macrophages underwent DNA fragmentation (4.6-fold increase in average MFI) as a consequence of infection (Figures 1A and B). Caspase activity was evaluated by a fluorogenic technique. Macrophages did not show caspase 3 activity as a result of *M. bovis* infection; caspase activation was measured 4, 8 and 16 h post-infection (Figure 1C).

**Figure 2:**
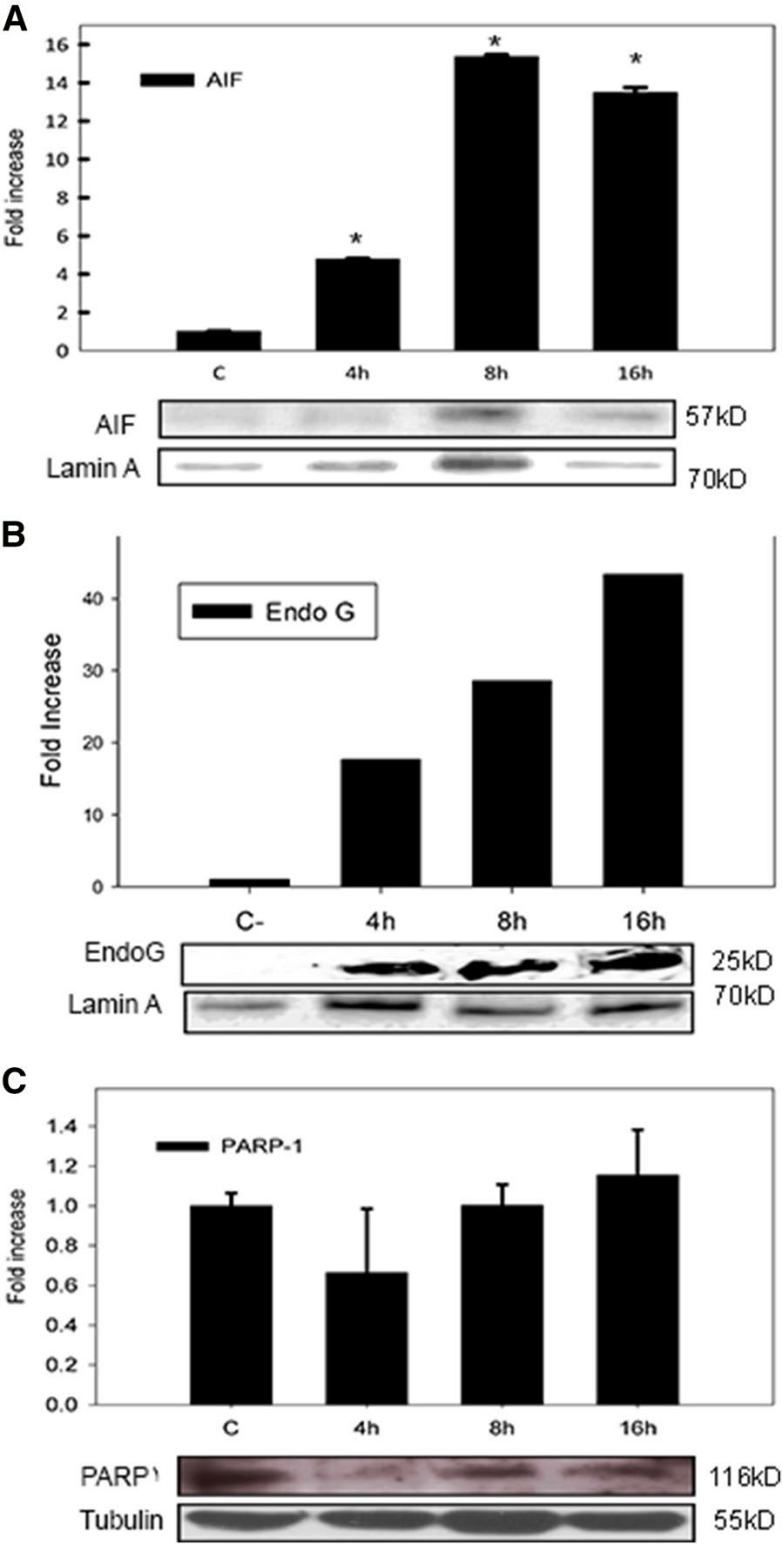
**AIF and Endo G are translocated to macrophage nuclei as a consequence of** ***M. bovis*** **infection.** Bovine macrophages were infected (MOI 10:1) with *M. bovis* AN5 strain at different time points (4, 8 and 16 h). Nuclear cell extracts from infected macrophages were analyzed by immunoblot, **A** AIF, **B** Endo G and **C** PARP-1. Protein content load was monitored with anti-Lamin A for nuclear extracts and anti-Tubulin for whole crude protein extracts. The results are representative of three independent experiments and are expressed as ratio of AIF and Endo G/Lamin A or PARP-1/Tubulin. Asterisk represents a p value equal or less than 0.05 of statistical significance.

Previous results from our group demonstrated apoptosis induction with AIF participation. However, AIF identification was accomplished by immunocytochemistry. In order to confirm this event, *M. bovis* infected and uninfected cells were analyzed by immunoblot. The results identified AIF presence in infected cell nuclei. Immunoblots showed 4.7, 15.3 and 13.49 fold increase in nuclear AIF at 4, 8 and 16 h post-infection respectively (Figure 2A). Our results confirmed AIF nuclear translocation as a consequence of *M. bovis* infection (Figure 2A).

### Nuclear concentration of Endo G but not PARP-1 is increased by *Mycobacterium bovis* infection

To determine whether other proteins associated to caspase-independent apoptosis were involved, we examined the presence of Endo G and PARP-1 in nuclear extracts of *M. bovis*-infected macrophages. Endo G concentrations in infected cells were approx 43-fold when compared to uninfected macrophages (Figure 2B). *M. bovis* induced a nuclear increase of Endo G and AIF, suggesting that the membrane potential of mitochondria could be disrupted by the infection. One of the release mechanisms of these proteins is associated with the production of Polymers of ADP-ribose by expression of PARP-1 in response to DNA damage. Therefore, we measured PARP-1 expression in the nuclei of cells infected with AN5; the results indicate that PARP1 does not have a differential expression due to infection (Figure 2C).

### Cyclosporine A treatment of *M. bovis*-infected macrophages decreased AIF and Endo G nuclear translocation and DNA fragmentation

**Figure 3:**
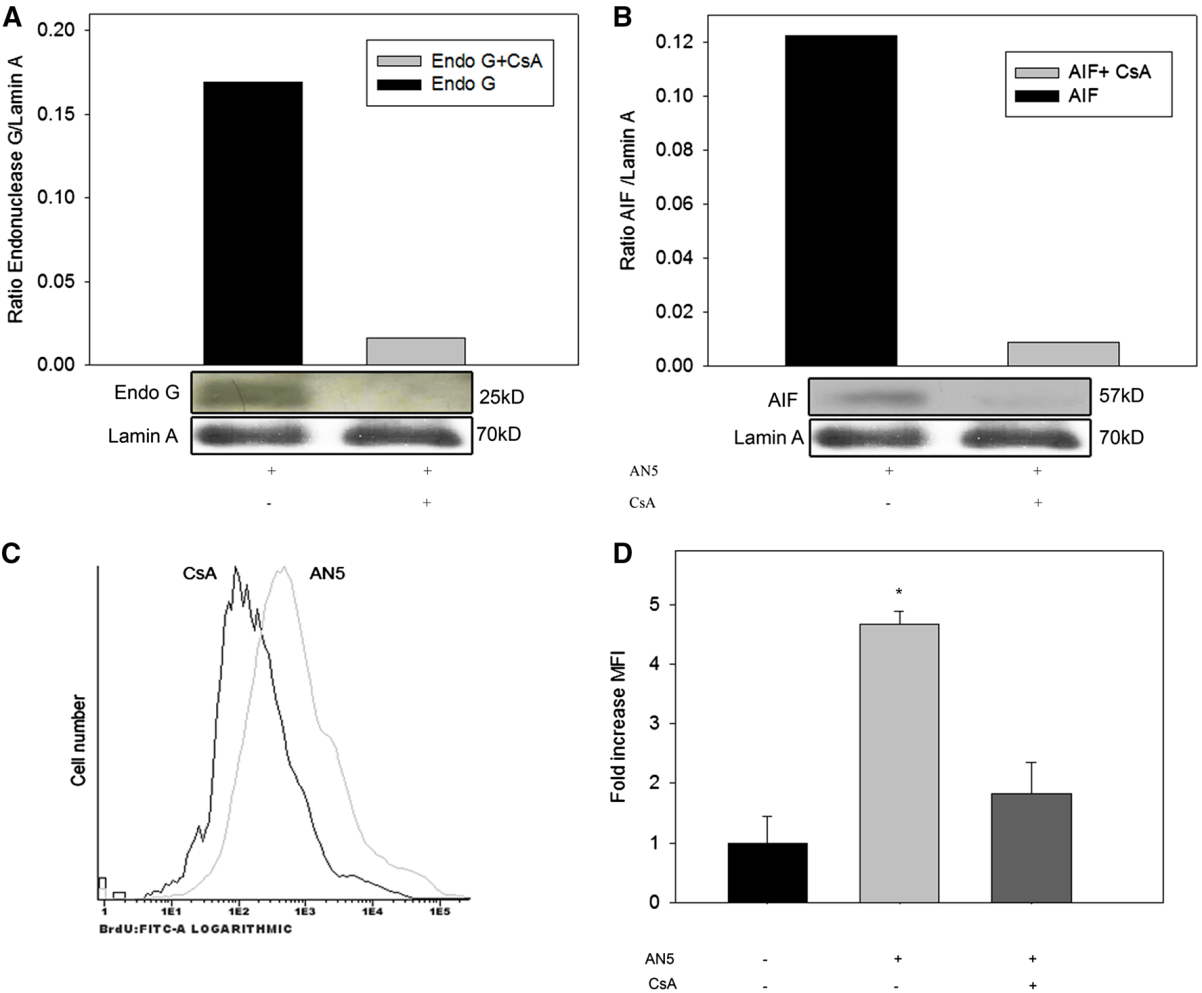
**Cyclosporine A (CsA) inhibits AIF and Endo G nuclear translocation and DNA fragmentation of** ***M. bovis*-infected macrophages.** Bovine macrophages were pretreated with 1 µM of CsA before *M. bovis* infection with AN5 strain (10:1) during 16 h. Concentration of **A** AIF, **B** Endo G was monitored by immunoblot. Protein content load was monitored with anti-Lamin A. The results are representative of two independent experiments and are expressed as ratio of AIF or Endo G/Lamin A. **C** DNA fragmentation was analyzed by TUNEL (BrdU-FITC), the results are representative of a FACS plot of TUNEL. **D** Quantification of DNA fragmentation in C, histogram shows mean fluorescence intensity (MFI) fold change of *M. bovis*-infected and CsA-treated macrophages with respect to uninfected cells. The results are representative of three independent experiments and are expressed as total cell counts versus FITC signal in log_10_ scale. One-way ANOVA shows significant variation in infected cells versus CsA-treated uninfected control (*p* < 0.05) but not between CsA and negative control (*p* = 0.16536).

Endo G and AIF are mitochondrial proteins involved in caspase independent apoptosis that are released after MOMP and translocated to the nuclei. In an attempt to verify the role of nuclear translocation of these mitochondrial proteins, we used Cyclosporine A (CsA) as an inhibitor of mitochondrial permeability transition and measured nuclear AIF and Endo G by immunoblot. CsA treatment of infected macrophages abolished nuclear translocation of AIF and Endo G (Figures 3A and B). Furthermore, the average MFI of TUNEL positive cells under these experimental conditions was very similar to non-infected cells (0.8 fold) (Figures 3A–D). Our results suggest that nuclear AIF and Endo G are associated with induction of DNA fragmentation; however, further investigation is required to confirm the association.

### *Mycobacterium bovis* intracellular survival is enhanced in the absence of AIF and Endo G nuclear translocation

**Figure 4:**
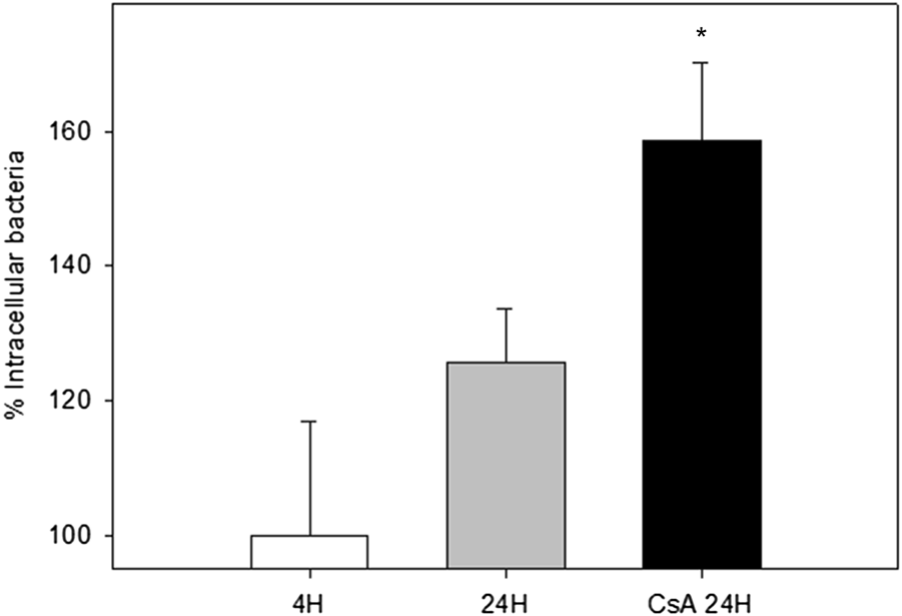
**Cyclosporine A treatment modifies the number of intracellular mycobacteria in bovine macrophages.** Macrophages were infected with *M. bovis* AN5 (MOI 10:1) for 4 h to allow phagocytosis, washed to remove extracellular bacteria and cultured again for 24 h in the presence or absence of Cyclosporine A. Bacterial proportional growth was calculated by dividing the number of intracellular CFU at 24 h post-infection by the number of intracellular CFU at the end of phagocytosis and expressed as percentage. Values are mean ± standard deviation from two independent experiments each one with three internal replicas. Asterisk represents a *p* value equal or less than 0.05 of statistical significance.

A microbicidal assay was performed to identify the effect of CsA treatment in bacterial intracellular survival. Bacterial colony forming units were quantified after plating live intracellular bacteria, released from Tween 20-treated macrophages. CsA treatment altered bacterial intracellular survival. CsA-treated macrophages show a 26.2% increase of viable bacteria 24 h after infection. CsA did not interfere in the viability of *M. bovis* AN5 (Additional file 1). These results suggest that apoptosis may function as a host protective response independently of caspase activity (Figure 4).

### PARP-1 inhibition does not modify apoptosis induction in *M. bovis*-infected macrophages

**Figure 5:**
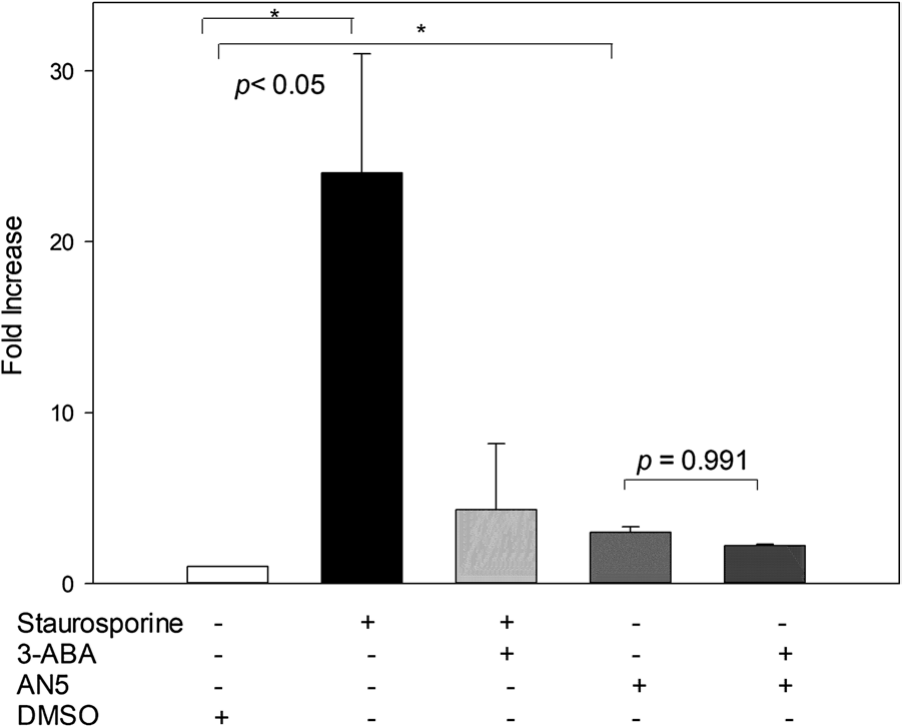
**DNA fragmentation of** ***M. bovis*-infected macrophages was not inhibited by 3-ABA treatment.** Bovine macrophages were pretreated with 50 µM of 3-ABA before *M. bovis* infection with AN5 strain (10:1) during 16 h. Quantification of DNA fragmentation shows mean fluorescence intensity (MFI) fold change of *M. bovis*-infected and staurosporine-treated (0.5 µM) macrophages with respect to uninfected cells. The results are representative of three independent experiments. Asterisk represent a *p* value equal or less than 0.05 of statistical significance.

In an attempt to identify PARP-1 contribution to apoptosis induction by *M. bovis* in macrophages, we preincubated *M. bovis*-infected bovine macrophages with 3-aminobenzamide (3-ABA), a PARP-1 inhibitor. A 2.9 and 2.2 fold increase in DNA fragmentation was observed in *M. bovis*-infected macrophages and *M. bovis* infected/3-ABA preincubated cells respectively compared to uninfected macrophages. The slight decrease in MFI associated with 3-ABA treatment was not statistically significant (*p* = 0.991), suggesting that PARP-1 does not play a role in *M. bovis* associated apoptosis (Figure 5). As a positive control, we used staurosporine, a known parthanatos inducer. DNA fragmentation was significantly decreased (5.55 fold decrease) by 3-ABA in staurosporine treated macrophages, validating this PARP-1 inhibitor.

## Discussion

The major finding of this paper is that inhibition of the release of mitochondrial proteins, including AIF and Endo G, blocks caspase-independent apoptosis induced by *M. bovis* in bovine macrophages. Our group reported for the first time that *M. bovis* induces apoptosis in bovine macrophages [22], we proceeded to demonstrate that apoptosis took place in the absence of caspase activation and suggested that AIF might be involved [18]. In this study we confirmed our earlier observations using a more reliable and accurate technique; we found a 15.3-fold (8 h) AIF increase in nuclear extracts of infected macrophages by immunoblot analysis, demonstrating that *M. bovis* infections trigger the release of mitochondrial proteins. In an attempt to gain additional insight into the identity of other mitochondrial cell death effectors, we searched for Endo G nuclear accumulation; our results indicate that soon after infection, not only AIF but also Endo G, is mobilized out of mitochondria to later on enter the nucleus. Our observations propose that *M. bovis* infection affects the mitochondrial outer membrane permeability. For this reason, we designed an experiment to corroborate this hypothesis. Pretreatment of *M. bovis*-infected macrophages with cyclosporine A (CsA), a mitochondrial permeability transition pore (MPTP) inhibitor, abolished AIF and Endo G nuclear translocation. In addition, it also decreased to baseline macrophage DNA fragmentation induced by the mycobacterial infection. Moreover, CsA treatment altered bacterial intracellular survival, inducing an increase of bacterial viability in 26.2%. In order to demonstrate that CsA did not alter *M. bovis* growth in vitro, we incubated *M. bovis* AN5 in RPMI for 24 h with or without CsA. Our results show that CsA did not interfere with bacterial growth. Altogether, our data suggest that one of the main effects of *M. bovis* infection in bovine macrophages is to induce MOMP, allowing the release of proteins, including AIF and Endo G, from the mitochondria, which translocate into the nucleus where their presence induces DNA fragmentation. Interestingly, our results also support the concept that caspase activity is not required to decrease Mycobacterial survival. When observing the bovine model, we can see that macrophage cell death occurs without caspase activation; we discarded activation of caspases 3, 8 and 9 [18], therefore it is likely that the caspase associated proteins cytochrome C and Smac/DIABLO are not released from mitochondria under our experimental conditions. Cytochrome C induces the oligomerization of Apaf-1 to form a caspase-activating apoptosome complex, whereas Smac/DIABLO participates by eliminating the caspase inhibitory effect of the inhibitor of apoptosis proteins (IAP) [23]. Evidently, our proposition is speculative at this stage and requires further investigation. AIF is a flavoprotein that functions as an electron transferase; it is also a cell-death regulator that induces chromatin condensation and DNA fragmentation by an unknown mechanism [24, 25]. However, it has been proposed that it might be responsible for DNA damage through free radical-mediated DNA cleavage, or by recruitment and activation of other factors like nucleases [24]. Endo G is an endonuclease that cleaves chromatin DNA into nucleosomal fragments [14]. Whether these mitochondrial proteins act independently or in combination to induce the DNA fragmentation observed in this study, or if other proteins are involved, is not known and additional research is required. The term caspase independent cell death (CICD) has been applied to cell demise occurring in the presence of pan-caspase inhibitors. CICD has been reported in cells exposed to Bax, TNFα, chemotherapeutic drugs, and bacterial toxins [26]. Infection by mycobacterial species may stimulate CICD as well [27–30]. Involvement of bacteria in caspase-independent apoptosis has been previously reported; some known examples are pathogenic *Leptospira* species and Group B *Streptococcus* that have been shown to induce caspase-independent macrophage apoptosis with Endo G participation [31, 32]. To the best of our knowledge, this is the first report associating Endo G with caspase-independent apoptosis induced by a member of the *Mycobacterium tuberculosis* complex. In this study we attempted to explore the possible role of PARP-1 in apoptosis induction within *M. bovis*-infected bovine macrophages. We demonstrated that PARP-1 protein expression in macrophages was not changed by *M. bovis* infection (Figure 2). In addition, pretreatment of *M. bovis*-infected bovine macrophages with 3-ABA, a PARP-1 inhibitor, did not change the proportion of macrophage DNA fragmentation (Figure 5). Collectively, our results propose that PARP-1 does not participate in macrophage apoptosis, therefore, it is possible to hypothesize that parthanatos is not taking place under these experimental conditions, even though we documented a high nitric oxide production that has been associated with DNA damage. Whether the release of AIF from mitochondria in macrophages infected by *M. bovis*, is induced by PAR or an alternative inducer, is unknown. Although we did not measure PAR concentration, we may speculate that PAR is not the only molecule associated with AIF release from mitochondria [15]. Apoptosis is considered to be a mechanism of innate immunity. Macrophage apoptosis is a host strategy against mycobacteria that plays a role in decreasing bacterial viability, preventing dissemination of the pathogen and promoting T cell cross priming by antigen-presenting cells [6, 33, 34]. On the contrary, apoptosis induction may be considered a mechanism useful for bacteria to avoid innate host defense. Virulent strains of *M. tuberculosis* induce more necrosis and less apoptosis than the attenuated ones [35–37]. In this scenario, it is plausible to speculate that the absence of caspase activation in *M. bovis*-infected macrophages may be a pathogen mechanism to evade the cell death process that would provide an intracellular niche required by bacteria to survive. However, macrophages respond using a counterattack maneuver that switches on an alternative cell death pathway, independent of caspase activation, thus maintaining an upper hand in this battle against bacteria. We used *M. bovis* AN5 strain to conduct our experiments, similar to all the strain tested in our laboratory, AN5 induced DNA fragmentation, confirming that it is a bacterial competence to induce apoptosis. Moreover, bovine macrophages incubated with an *M. bovis* protein extract also developed structural changes compatible with apoptosis [18]. Lastly, preliminary results from our laboratory have shown that individual mycobacterial proteins are also capable of inducing apoptosis without caspase activation. Collectively, this data demonstrates that *M. bovis* and its protein components prompt bovine macrophages to undergo apoptosis in a caspase-independent fashion. In this paper, we further dissect this apoptotic pathway; our data support the central role of mitochondria in this event and points out AIF and Endo G as two possible molecular executioners.

## Additional file

**Additional file 1.** **CsA does not affect** ***Mycobacterium bovis*** **growth in vitro.** We incubated 1 × 10^5^ mycobacteria in RPMI during 24 h with or without CsA, bacterial growth was calculated by plating serial dilutions and CFU counting.
