# Supplementary figures and images for: Endonuclease G takes part in AIF-mediated caspase-independent apoptosis in Mycobacterium bovis-infected bovine macrophages
Source: Vet Res. 2018 Jul 18;49:69. doi: 10.1186/s13567-018-0567-1 (PMC6052627; doi:10.1186/s13567-018-0567-1)

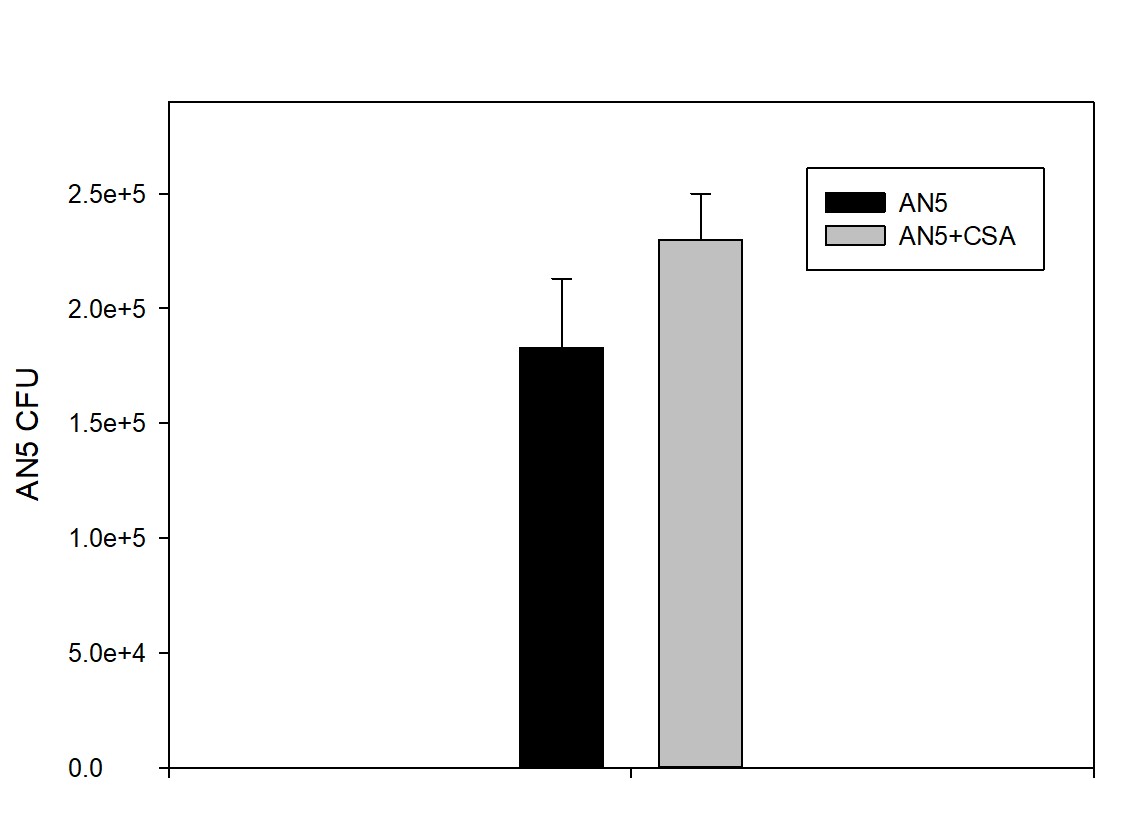

Supplement: Supplementary file 1 — Additional file 1. CsA does not affect Mycobacterium bovis growth in vitro. We incubated 1 × 10^5^ mycobacteria in RPMI during 24 h with or without CsA, bacterial growth was calculated by plating serial dilutions and CFU counting. [file 13567_2018_567_MOESM1_ESM.jpg]
